# New Targets for Drug Discovery against Malaria

**DOI:** 10.1371/journal.pone.0059968

**Published:** 2013-03-28

**Authors:** Guido Santos, Néstor V. Torres

**Affiliations:** 1 Departamento de Bioquímica y Biología Molecular, Universidad de La Laguna, San Cristóbal de La Laguna, Tenerife, Spain; 2 Instituto Universitario de Enfermedades Tropicales y Salud Pública de Canarias, Universidad de La Laguna, San Cristóbal de La Laguna. Tenerife, Spain; Instituto de Ciências Biomédicas/Universidade de São Paulo - USP, Brazil

## Abstract

A mathematical model which predicts the intraerythrocytic stages of *Plasmodium falciparum* infection was developed using data from malaria-infected mice. Variables selected accounted for levels of healthy red blood cells, merozoite (*Plasmodium* asexual phase) infected red blood cells, gametocyte (*Plasmodium* sexual phase) infected red blood cells and a phenomenological variable which accounts for the mean activity of the immune system of the host. The model built was able to reproduce the behavior of three different scenarios of malaria. It predicts the later dynamics of malaria-infected humans well after the first peak of parasitemia, the qualitative response of malaria-infected monkeys to vaccination and the changes observed in malaria-infected mice when they are treated with antimalarial drugs. The mathematical model was used to identify new targets to be focused on drug design. Optimization methodologies were applied to identify five targets for minimizing the parasite load; four of the targets thus identified have never before been taken into account in drug design. The potential targets include: 1) increasing the death rate of the gametocytes, 2) decreasing the invasion rate of the red blood cells by the merozoites, 3) increasing the transformation of merozoites into gametocytes, 4) decreasing the activation of the immune system by the gametocytes, and finally 5) a combination of the previous target with decreasing the recycling rate of the red blood cells. The first target is already used in current therapies, whereas the remainders are proposals for potential new targets. Furthermore, the combined target (the simultaneous decrease of the activation of IS by gRBC and the decrease of the influence of IS on the recycling of hRBC) is interesting, since this combination does not affect the parasite directly. Thus, it is not expected to generate selective pressure on the parasites, which means that it would not produce resistance in *Plasmodium*.

## Introduction

According to the World Health Organization [1], malaria affects more than 500 million people worldwide, killing between 1 and 2.5 million people annually, most of whom are children under the age of five. It is caused by *Plasmodium* genus parasites (*Plasmodium vivax, P. ovale, P. malariae, P. knowlesi* and *P. falciparum*), *P. falciparum* being the most lethal. The parasites multiply inside human erythrocytes, killing the cells in the process, and are transmitted by female *Anopheles* mosquitoes. The area most affected by malaria is sub-Saharan Africa.

There is currently no effective vaccine against malaria. Some promising preliminary results have been seen, but no solution to this issue is expected over the next few years [2]. To make the situation even worse, the efficacy of transmission control by means of insecticide-treated nets and indoor residual spraying is dropping, because resistance to insecticides is increasing among mosquitoes in Africa [3]. Because of that malaria control is becoming totally dependent on pharmacological treatments.

There are several classes of drugs used to treat malaria. All share the feature of targeting the merozoites [4], [5], while some target gametocytes as well. These drugs include quinolines, antifolates and artemisinin, administered alone or in combination. Quinolines are thought to affect the polymerization of hemozoin, which is toxic to the parasite. Antifolates inhibit the synthesis of folic acid by blocking the dihydrofolate reductase and dihydropteroate synthetase enzymes of the parasite. Although the mechanism of action of artemisinin is not known, the most accepted one is interference with the plasmodial sarcoplasmic/endoplasmic calcium ATPase [4]. Resistance to all these antimalarial drugs has been widely reported [5], even in the case of what the World Health Organization has identified as the most effective treatments, the artemisinin combined therapies. In particular, some resistance to the artemisinin combined therapies has been detected in South-East Asia. This poses a potentially dangerous and severe scenario, if the resistance spreads to endemic areas in Africa [6], [7] since, to our knowledge, no other effective antimalarial treatments are in sight.

This situation can be attributed, at least in part, to the classical, reductionist pharmacological approach to finding new drugs. This approach is mainly based on reducing the disease to a small set of defined targets for which new drugs can be sought. In the case of malaria, it is evident that this approach has shown little success, a trend also observed in other complex diseases [8].

Wells 2010 divides all current drug discovery strategies into two groups: “whole parasite screening” and “rational design approaches” [9]. Whole parasite screening strategies are based on testing compounds *in vitro* and selecting those which affect a *Plasmodium* culture. The rational design approach strategies try to inhibit specific pathways of the parasite. Screening approaches have the limitation that they are non-directed; there are a huge number of possible compounds to test and the entire screening process is conducted with the parasite isolated from the host system in *in vitro* conditions. Rational design is directed, but it depends on the knowledge of the mechanisms of the parasite [9] and is thus very reductionist in focus. Furthermore, none of these methods can deal with the existing pharmacological targets which only work *in vivo.*

While due credit must be given to these reductionist approaches for their contribution to the development of the drugs currently available, it is not equally clear that this strategy has proven sufficiently effective in providing a number of relevant drug solutions [10].

Accordingly, a third line of approach has been proposed to direct drug discovery [11]. This strategy, known as quantitative and systems pharmacology, aims to understand how drugs influence cellular networks in space and time and determine how they affect human pathophysiology. This approach follows the tenets of systems biology, and is based on the development of mathematical models that incorporate data at several temporal and spatial scales and are able to predict the dynamic behavior of the main variables involved in the parasite infection and the therapeutic effects of drugs. The principles of system biology thus provide the methodological framework and perspective needed for modeling system behavior *in vivo*, establishing the basis for a genuinely rational target identification and drug design. In this context, the defining feature of system biology is the combined use of mathematical and computable models and quantitative experimental data as a means to unravel the network-based (“emergent”) properties which cannot be deduced in any way from the knowledge of their components [12].

Such models, which lead to an educated and informed hypothesis, can then be used to identify the most sensitive processes of the system with a view to reducing the parasitemia (see Länger et al, 2012; [13]). The present work is in line with this approach, and with the current view that in order to identify new and better targets for antimalarial drug-based treatments, a model-based approach is needed [11].

In the present case, a model system constructed in this way will represent *Plasmodium* infection *in* *vivo*, and thus it is to be expected that well-selected interventions in some of these processes in the real system will lead to a recovery in the patient. The results thus obtained will be correlated and compared with current therapies against malaria.

## Results

In order to analyze the dynamics of the infection process by *Plasmodium* and to propose new targets with potential for drug discovery against malaria, an ordinary differential equations mathematical model was developed (see Material and Methods). In the present study, we used the General Mass Action within the Power Law formalism [14]. This formalism has been used as the framework for modeling other infectious diseases [13]. Regarding other diseases, the range of pathologies that have been addressed using the Power Law formalism, either in the GMA or in the S-system version, goes from the analysis of the purine metabolism in human [15], [16] to inflammation and preconditioning [17], [18], mental disorders [19] and cancer [20].

The model so constructed is a phenomenological one, where some aspects of the physiological processes are lumped together and represented by a single process. The proposed model is focused on a critical phase of the *Plasmodium falciparum* life cycle within the host, namely the processes involved after the release of liver cell merozoites into the bloodstream.

**Figure 1:**
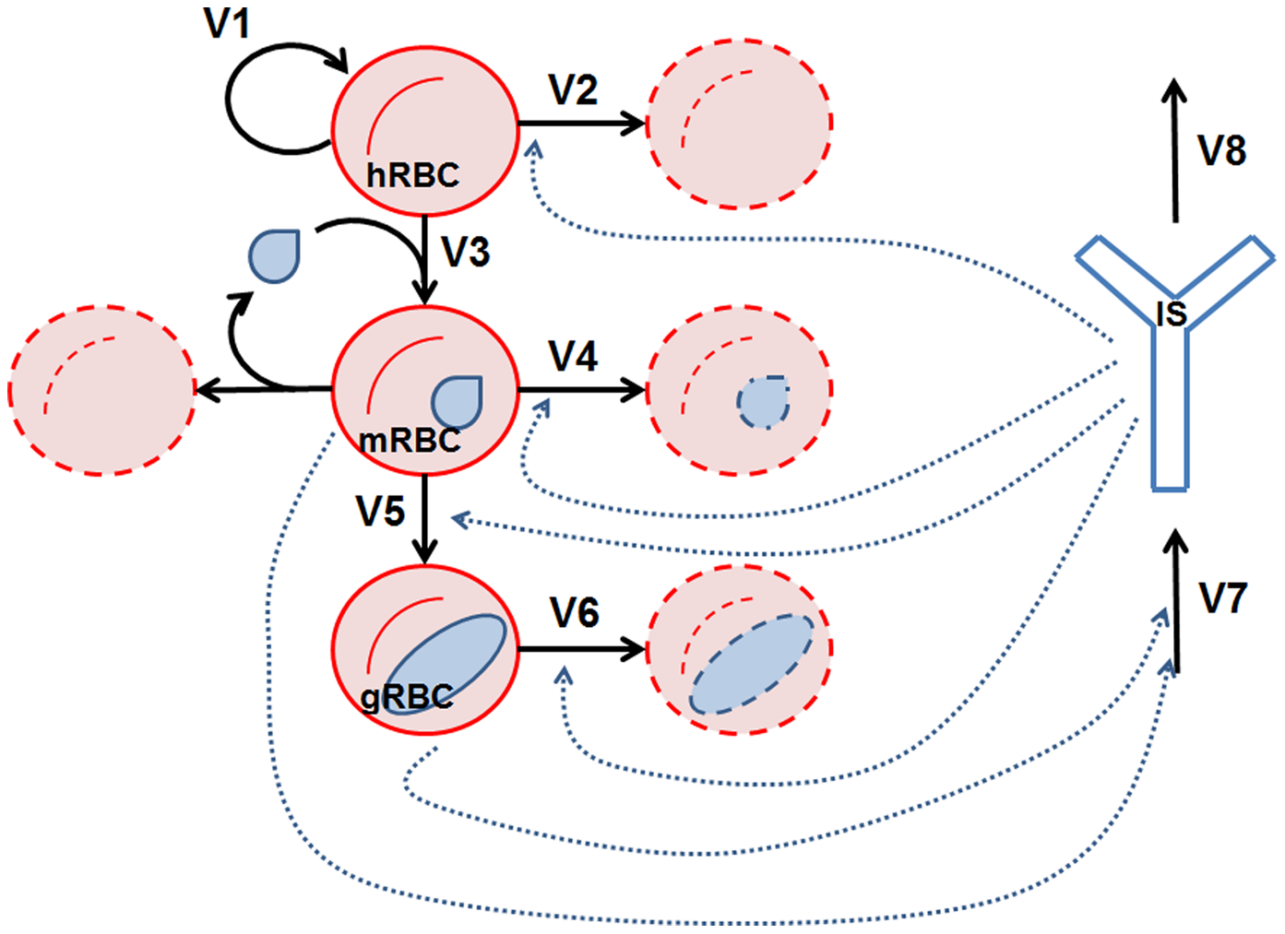
Erythrocyte infection model representation. In the picture, red circles represent the red blood cells, blue drops represent merozoites, blue ovals represent gametocytes and the blue “Y” represents the immune system. The variable acronyms are the healthy red blood cells (hRBC), the merozoite-infected red blood cells (mRBC), the gametocyte-infected red blood cells (gRBC) and the immune system (IS). Black continuous arrows represent processes while blue dashed arrows represent regulatory influences of the variables on processes.

As is well known, once they are in the bloodstream, the merozoites infect red blood cells, which in turn produce further merozoites. Subsequently, sexual forms (gametocytes) are produced which eventually, if taken up by a mosquito, will infect the insect and will continue the parasite life cycle. Figure 1 shows the simplified representation of these processes. The selected variables include the healthy erythrocyte population (hRBC) and the merozoite-parasitized erythrocyte cell density (mRBC). This latter variable represents all red blood cells infected at whatever stage of the asexual cycle of the merozoite. Another variable is the gametocyte-parasitized erythrocytes (gRBC), which represents all red blood cells containing a *Plasmodium* gametocyte. These three variables are all expressed as units per volume of blood (µL). A final variable accounts for the overall activity of the immune system (IS). The IS is a phenomenological variable measuring the mean response of the immune system against *Plasmodium* infection. The mouse IgG1 (immunoglobulin G1) measured in absorbance units is taken to represent the activity status of the immune system.

In the figure, black continuous arrows represent processes actually occurring during this stage of the infection, while blue dashed arrows stand for positive regulatory influences of the variables on different processes. In our model, the different stages of the intraerythrocytic cycle of parasite are represented by (mRBC; merozoites) and (gRBC; gametocytes). The processes represented in the model include the synthesis of hRBC by the host (V1), balanced by a rate that integrates the natural processes leading to erythrocyte decay (V2); the immune system (IS) has an influence on this rate [21]. Healthy erythrocytes are infected by *Plasmodium* merozoites, a process that is stimulated by the growing population of infected erythrocytes (mRBC) (V3) [22]. The transformation of mRBC into gRBC is also considered (V5). This process is assumed to be influenced by the IS (a representative component of the parasite stress in the model; see Material and Methods section), as has been shown [23]. The elimination of the infected red blood cells (mRBC and gRBC) is included (V4 and V6, respectively); this elimination is promoted by the IS. The model integrates all processes related to the activation of the immune components controlling the malaria parasite inside the host through the activation of the variable IS (V7). Finally, the inactivation of the IS is included (V8). In the model, we assume that the process leading to the growth of the free parasite population inside the liver does not affect its dynamics at the intraerythrocytic level.

### Model Validation

**Figure 2:**
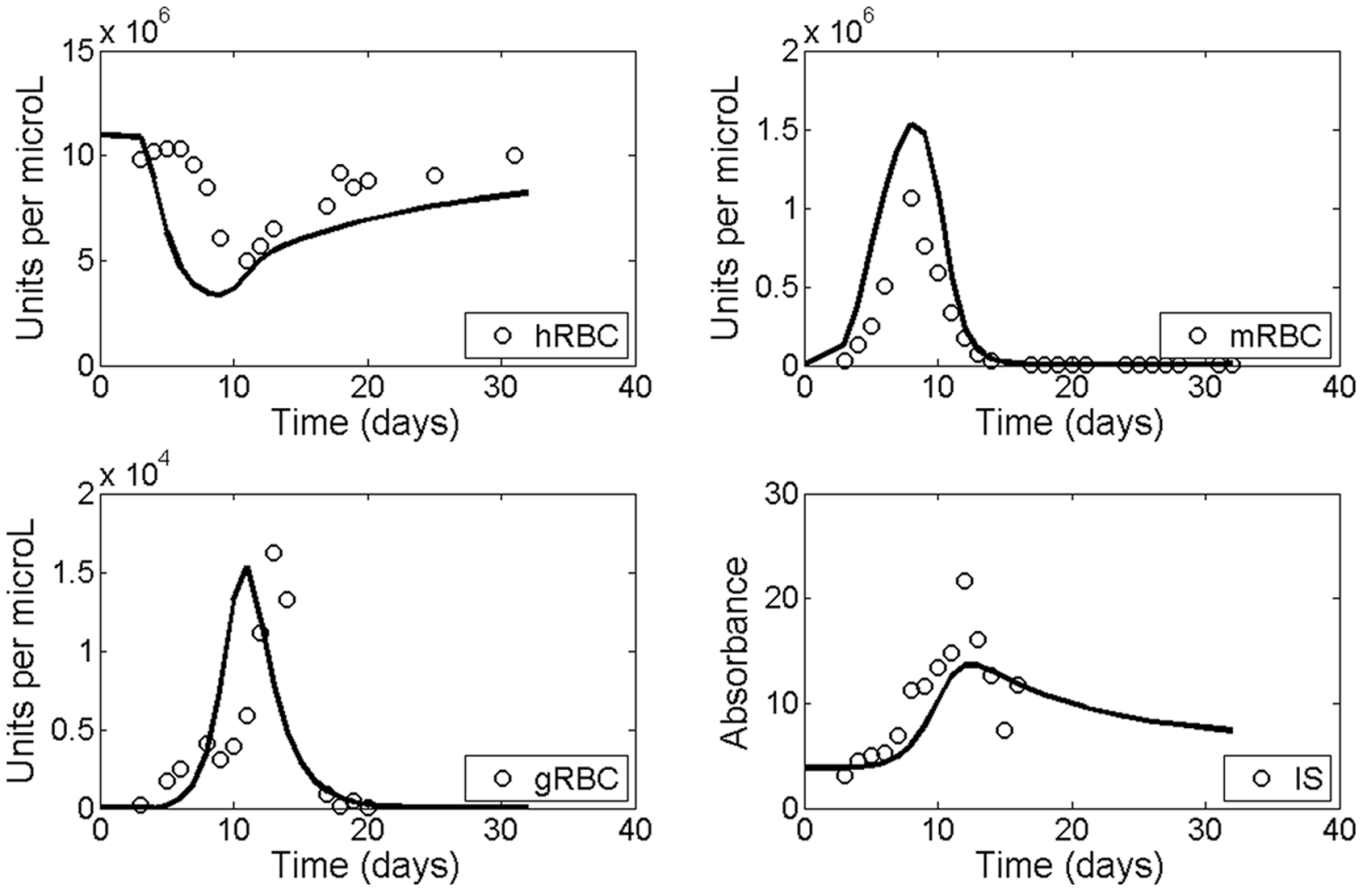
Model fitting. In all panels, continuous lines represent the model’s prediction while dots represent the experimental data obtained from *Plasmodium chabaudi* [24], [25]. Variable values are normalized.

Figure 2 shows the comparison between the model’s fitting and the experimental data. Experimental measures were taken from the bibliography [24], [25] and consist of measures from *Plasmodium chabaudi*-infected mice of different malaria-related components (healthy red blood cells, merozoite and gametocyte-infected red blood cells and IgG1). The initial conditions for the variables hRBC and IS, since they should represent the system condition before infection, were taken from the first experimental values in Figure 2. For all validations the values of the parameters were kept fixed.

Before the model is utilized for our intended purposes, it should be diagnosed for internal robustness. Accordingly, we identified and evaluated the stability of the initial and final steady states and carried out the sensitivity analysis (see Figure S1). Moreover, given the dynamic nature of the process, we calculated the dynamic sensitivities that serve to assess how changes in initial values affect the transient responses of a system (see Figure S2). Furthermore, for a model to be reliable and useful, its predictions should be validated against the performance of the actual system in different experimental conditions. For this purpose, we used three different sets of experimental data obtained in diverse experimental settings and conditions. First, we compared the model’s predictions with data on the dynamics of *Plasmodium falciparum* parasitemia in human hosts; secondly, experimental time series data of *Plasmodium falciparum* parasitemia in vaccinated and non-vaccinated monkeys were evaluated against the model’s predictions in correlative *in silico* scenarios; and thirdly, the observed dynamics of *Plasmodium chabaudi* in groups of drug-treated mice were contrasted with the model’s expectations. It should be stressed that in all cases, the experimental data used were obtained in different conditions and settings from those used when constructing the model.

#### Dynamics of Plasmodium falciparum in human host

**Figure 3:**
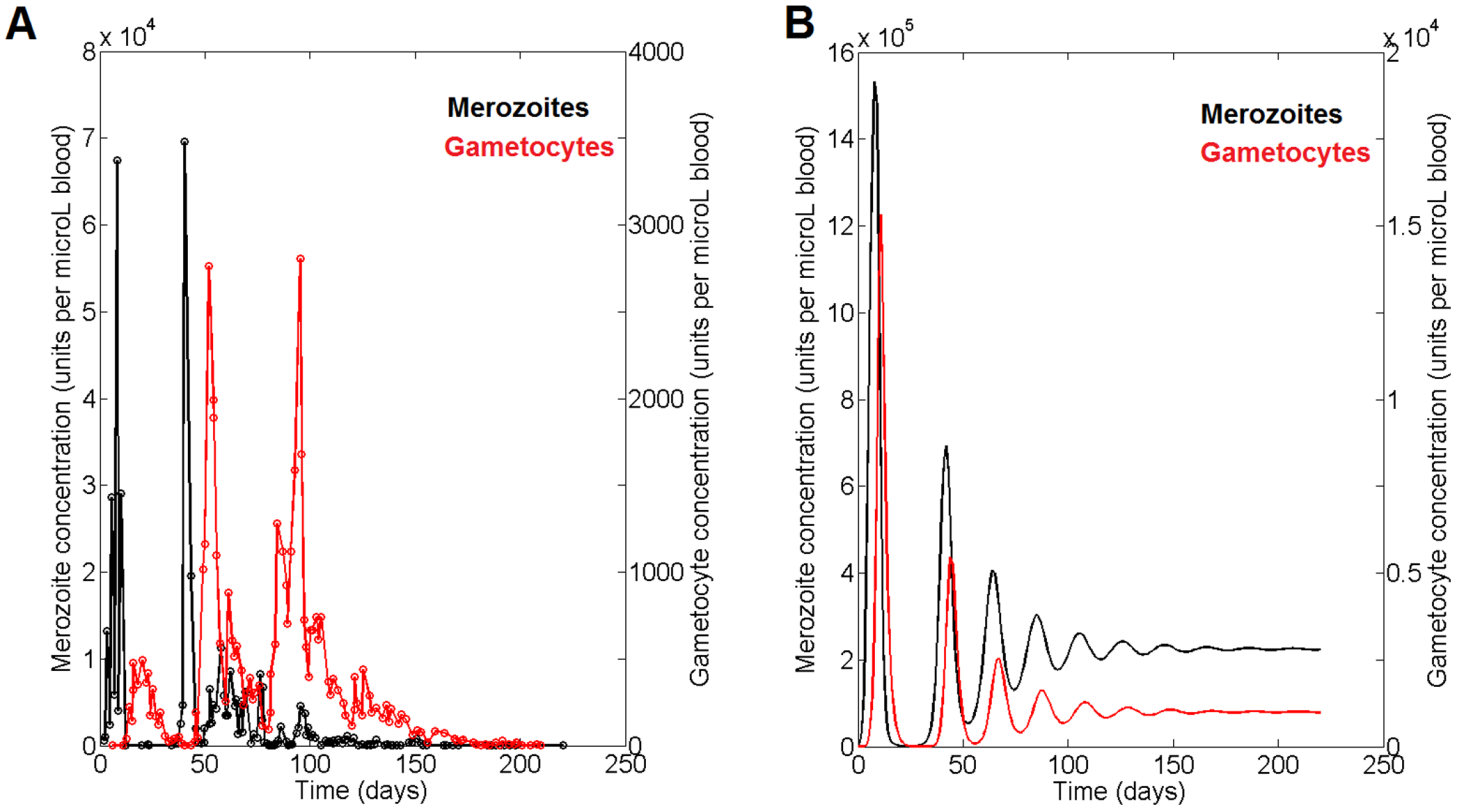
Dynamic prediction. **A.** Dynamics of the merozoites (black line) and gametocytes (red line) of *P. falciparum* measured in humans as described by Diebner et al. 2000 [26]. **B.** Prediction of the model on the dynamics of the merozoites (mRBC; black line) and gametocytes (gRBC; red line); variable values were normalized.

The first line of model validation was obtained from the comparison of the model’s predictions with the experimental data obtained by Diebner et al. 2000 [26]. These authors measured the dynamics of the concentration of *P. falciparum* merozoites and gametocytes in infected, non-treated humans. We compared their results with the model’s predictions (see Figure 3). From the observation of Panels A and B in Figure 3, a number of features emerge that merit further comment. The first observation is that the oscillating dynamics of the experimental parasitemia are reproduced by the model. More specifically, it should be noted that, although the experimental system (*P. falciparum* in infected, non-treated humans) differs from the one used to construct the model (*Plasmodium chabaudi* infection in mice), the model’s prediction of the merozoite and gametocyte population dynamics correlates well with the experimental measures in terms of both the magnitude of the period and the relative amplitude of the oscillations. Moreover, the model reproduces the later attenuation of the parasitemia. It can be observed, however, that although the first peaks of merozoites and gametocytes are reproduced well by the model, the second peak of merozoites occurs between the gametocyte peaks, which are not the case in the model predictions. Regarding this point, we should stress here that since our model is a phenomenological one, we do not expect to see a quantitative reproduction of the data. On the contrary, we should take into account the fact that, in spite of the fact that the model is based on observations made in mice infected with *P. chabaudi*, it is able to qualitatively reproduce the dynamics observed in a different host (human) infected with a different pathogen (*P. falciparum*). Since the kinetics of the life cycle differs in the new conditions, we consider that the dynamic qualitative pattern reproduction was good enough for our objective.

#### 2. Response to vaccination

**Figure 4:**
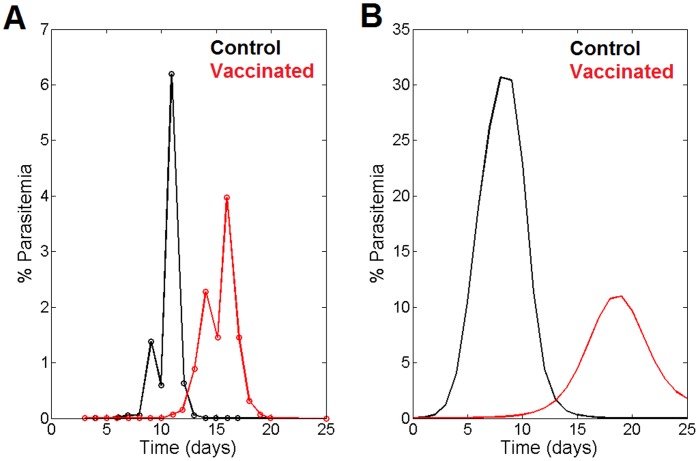
Experimental verification of the response to vaccination. **A.** Dynamics of the merozoites in non-vaccinated monkeys (black line) and in vaccinated monkeys (red line) of *P. falciparum* as measured by Makobongo et al. 2006 [27]. **B.** Prediction of the model on the effect of vaccination. Black line, control situation; red line, vaccinated condition. The vaccinated condition was represented in the model by increasing the initial value of the variable IS, thus mimicking an improved immunological status.

Additional evidence of the model’s quality comes from the comparison of the model’s predictions with experimental time series data obtained in a study carried out in vaccinated and non-vaccinated monkeys (Makobongo et al. [27]). Figure 4 compares the model’s predictions with the data on parasitemia dynamics obtained from non-vaccinated and vaccinated monkeys. Panel A shows the changes observed experimentally in parasitemia (merozoites) after vaccination, reflecting a delay in the maximum peak of parasitemia. In Panel B, the vaccinated condition was represented in the model by increasing the initial value of the variable IS, thus mimicking an improved immunological status**.** It can be seen that in spite of the fact that we are using a model based on the observed infection by *P. chabaudi* in CBA to predict the *P. falciparum* parasitemia dynamic in monkeys, the model is still able to reproduce both the general trend observed in the actual data and the specific features of the observed dynamics, such as the delay in the onset of the parasites and the time course of the parasitemia towards zero after 23 days. The difference in the % of parasitemia between Panels A and B can thus be attributed to the different experimental set-ups and to the well-known differences in the life cycle lengths of the parasite in monkeys and mice.

Taken together, these observations, based on different experimental set-ups and host types, lend support to the reliability of the model for its use as a testing bench in the search for effective antimalarial targets.

### Model Extension: Effect of Different Modes of Drug Administration

The model presented above can be extended in order to give a better interpretation of how the different modes of administration of a current drug affect the infection dynamics. The case is illustrated by the comparison of the model’s predictions with the course observed following certain antimalarial treatments. Chimanuka et al. [28] published the parasitemia time data series obtained following treatment of infected mice with the antimalarial drug β-artemether, which is known to enhance the death rate of the merozoites (mRBC). They studied the time course under two conditions: direct injection of the drug into the bloodstream and also when liposomes were used as a means for slowing drug delivery, which has been shown to yield better results measured as longer elimination half-lives.

**Figure 5:**
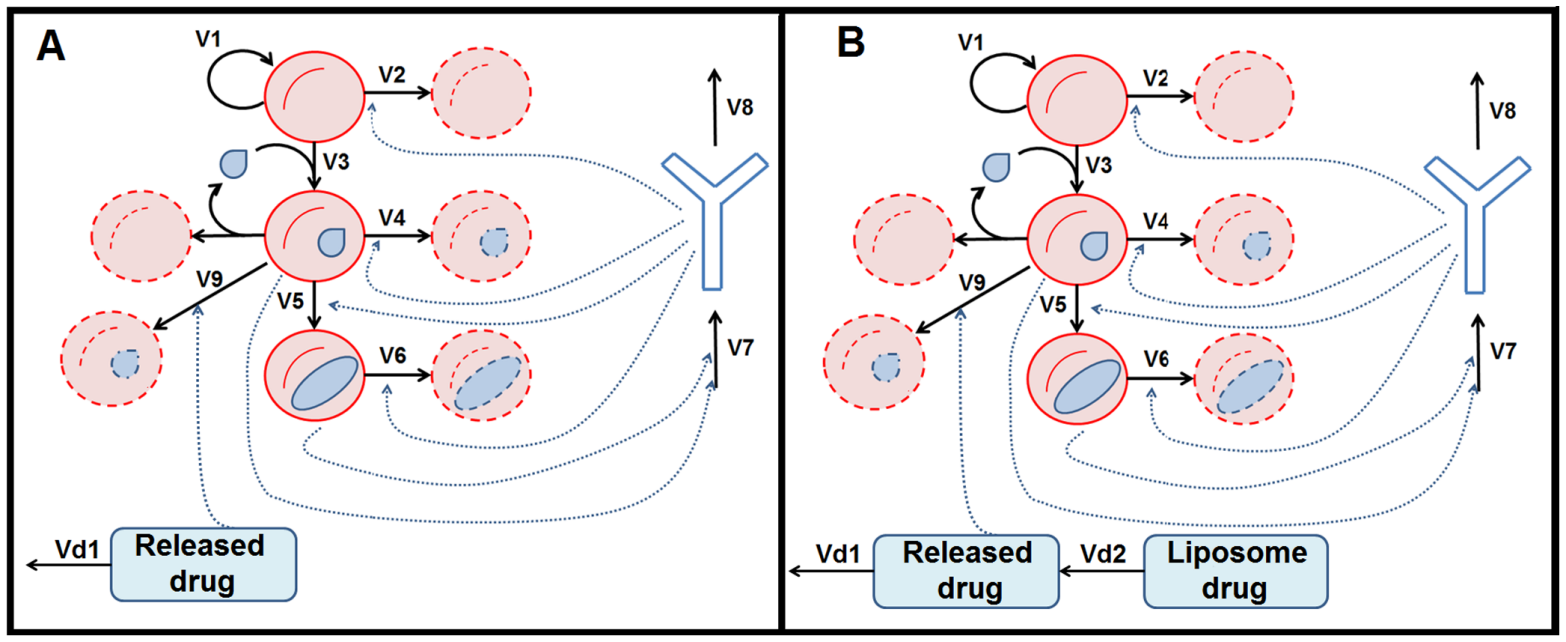
Model adaptation to treatment with antimalarial drug according with Chimanuka et al. [28]. **A.** The antimalarial drug is inoculated directly into the bloodstream. **B.** The drug is inoculated inside liposomes, which delays release into the bloodstream.

Equations 5 in the Material and Methods section shows the model changes made to represent the two drug delivery systems used by Chimanuka et al. [28] (see Figure 5). It is considered that β-artemether concentration linearly eliminates the parasite at the level of mRBC, through a process represented by V_9_. It can be argued that β-artemether would also affect V_4_. In fact, we explored this mechanism of action, but the best correlation with the experimental data by Chimanuka (data not shown) was obtained instead when a different process (V_9_) was assumed. This finding constitutes in itself a model prediction and suggests that the drug acts by eliminating the mRCB through a process different from its natural decay.

**Figure 6:**
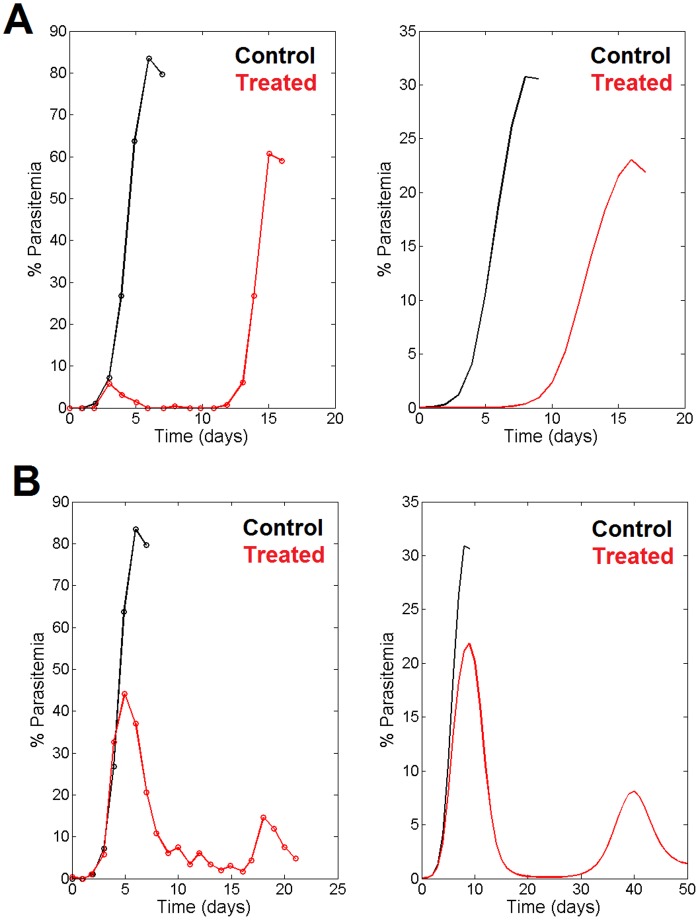
Experimental comparison of the model adaptation to treatment with antimalarial drug. **A.** When the antimalarial drug is inoculated directly into the bloodstream (Figure 5A), the left panel shows the experimental measure of merozoite parasitemia while the right panel shows the prediction of the merozoite parasitemia. Black line, parasitemia without treatment; red line, parasitemia under drug inoculation. **B.** When the drug is inoculated inside liposomes, the left panel shows the experimental measure of merozoite parasitemia while the right panel shows the prediction of the merozoite parasitemia. Black line, parasitemia without treatment; red line, parasitemia under drug inoculation. Data from Chimanuka et al. [28].

The comparison with the model’s parasitemia predictions is shown in Figure 6. Panel A shows the results of the simulation of the direct, free drug inoculation into the mouse’s bloodstream (right panel) compared with the experimental data (left panel). Panel B shows the simulation corresponding to drug delivery through the inoculation of liposomes carrying the drug into the bloodstream (right panel) compared with the experimental measures (left panel). In both cases the non-treated situation is shown in black. The temporal differences from Figures 6B with respect 6A are due to the different drug inoculation methods used in each case. Since the liposome drug release to the blood stream is slower, the dynamics takes more time.

In this figure it can be seen that the model’s predictions of the pattern dynamics of the infection correlate well with all three sets of experimental data. In the non-treated mice, the model reproduces, for the two conditions assayed (panels A and B) the experimentally observed initial peak of parasitemia. Also, the model reproduces the experimental observations of a low and delayed peak of parasitemia when the mice are treated by direct inoculation of the drug (panel A). Finally, the model’s prediction in conditions of intra-liposome drug inoculation (panel B) shows, in agreement with the experimental observations, a reduced initial peak of parasitemia followed by another, lower peak. In all cases, the model shows the oscillatory behavior as well as the relative decrease in the amplitude of the oscillations and the temporal delay between each. However, some discrepancies relative to the magnitude of the parasitemia in both panels, and the time scale in the Panel B are also evident. These differences can nevertheless be attributed to the fact that we are using a model based on the observed infection by *P. chabaudi adami* DK, a slow-growing strain [24] compared with the *P. chabaudi chabaudi* strain used for the verification data.

### Model-guided Search for New Drug Action Targets

Using this model as a basis, we applied various search routines (see Material and Methods) to identify antimalarial targets for the minimization of parasite load and response time after infection [29]. We carried out this search in two phases. We searched for “single-target solutions” first, and then for combinations of “two-target solutions”. The former aim at single targets, or processes that after modulation of their rate (either positively or negatively) by any means (e.g. by a drug) will cause the desired effect of reducing the parasite load and/or system response time. The latter target the same effects, but with consideration for the simultaneous modulation of two processes.

**Figure 7:**
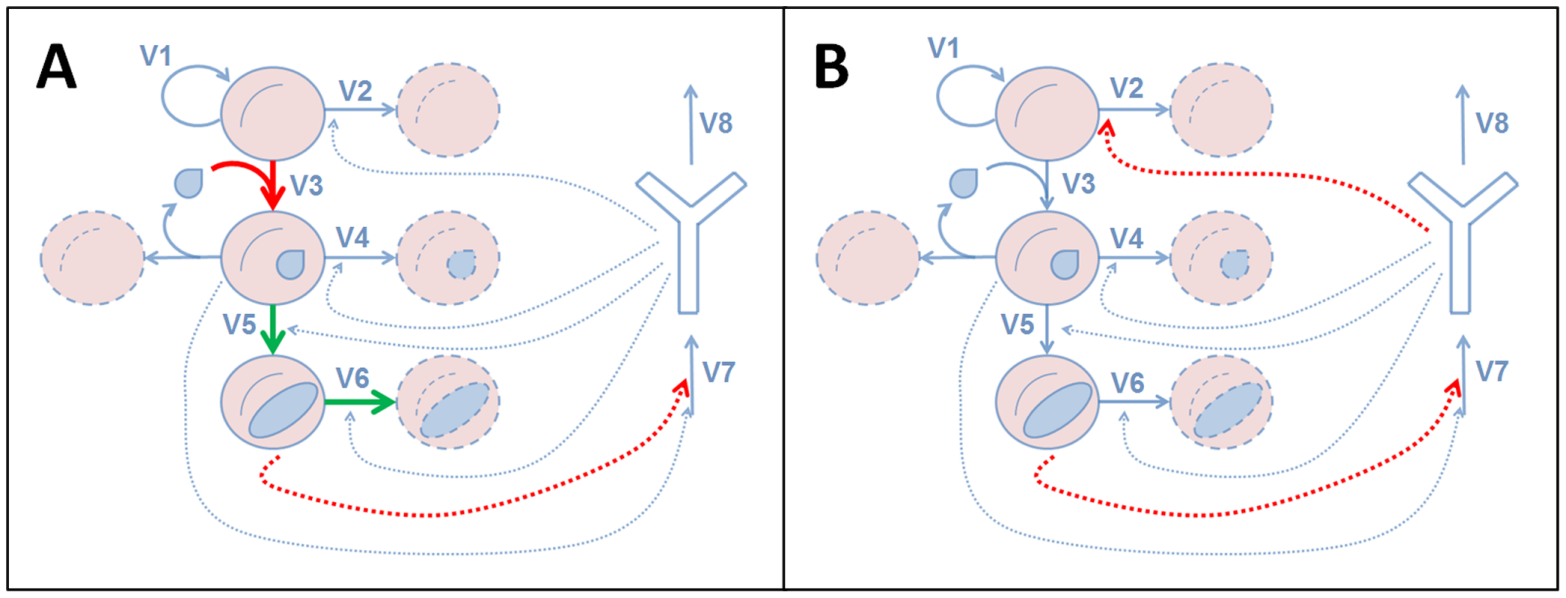
Single and combined targets for antimalarial drugs. **A.** Single targets (from top to bottom): decreasing invasion of hRBC (thick red arrow); increasing transformation of mRBC (thick green arrow); increasing death of gRBC (thick green arrow) and decreasing activation of IS by gRBC (dashed red arrow). **B.** Combined target: decreasing activation of IS by gRBC combined with decreasing recycling of hRBC (dashed red arrows).

The results obtained are summarized in Figure 7. It can be seen that there are four single-target processes (Panel A) and only one combination of two-target processes (Panel B) where modifications through the action of any drug or other agent can achieve a significant reduction in the parasitemia of the host.

In Panel A, we see a first (single) target, which consists of decreasing on the invasion rate of the red blood cells by merozoitos. The invasion of red blood cells involves the participation of a moving junction formed between the erythrocyte and the parasite membranes [30], [31]. The predicted effective intervention leading to a parasitemia reduction should be a reduction of this process. The other single targets detected involve the increase of the transformation rate of the merozoites into gametocytes (transformation of mRBC), the increase of the elimination of the gametocytes (death of gRBC) and the reduction of the influence of gametocytes on the synthesis and activation of the immune system (activation of IS by gRBC). In Panel B, we see the combined target predicted by the model, which consists of reducing the influence of gametocytes on the activation of the immune system (activation of IS by gRBC) while at the same time decreasing the elimination of healthy red blood cells by the immune system (recycling of hRBC).

**Figure 8:**
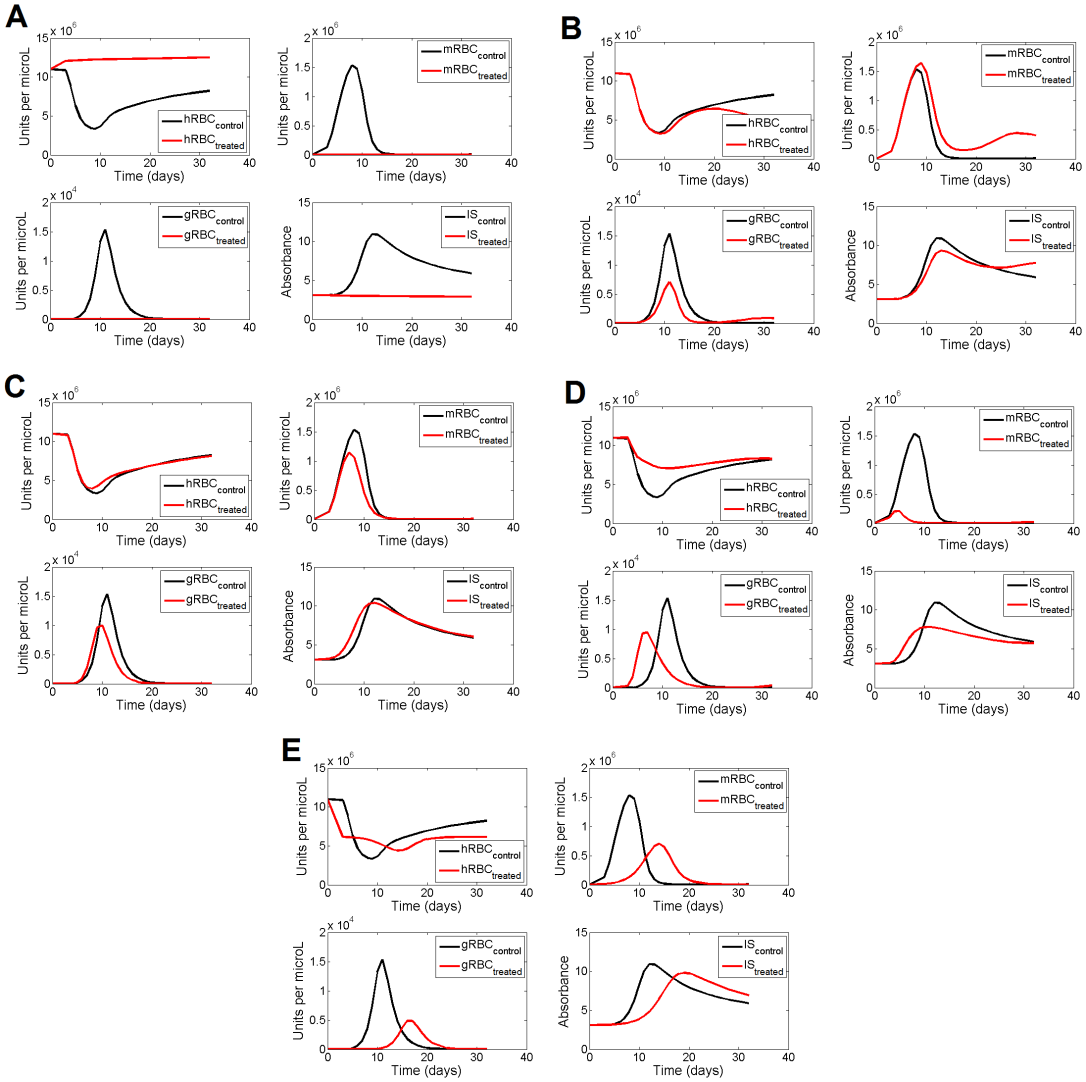
Model-based time course of the system variables after intervention on the selected target rate process. **A.** After a decrease of V_3_ (γ_3_, 25 times the initial, reference steady state value). **B.** After an increase of V_6_ (γ_6_, 3 times). **C.** Decrease of the influence of gRBC on V_7_ (g_73_, 2 times). **D.** Increase of V_5_ (γ_5_, 80 times). **E.** Simultaneous decrease of the influence of gRBC on V_7_ (g_73_, 2 times) and of the influence of IS on V_2_ (g_24_, 200 times). Continuous lines represent the prediction of the model in control conditions (no intervention on the target rate processes), while red continuous lines represent the prediction of the model after the corresponding interventions.

The results of the above interventions are summarized in Figure 8. Figure 8A shows the effects of reducing the invasion of hRBC (dividing by 25 the basal, reference value of γ_3_), thus mimicking the action of an antimalarial drug. It can be seen that the host variables hRBC and IS recover quickly and completely to healthy values, while mRBC and gRBC disappear quickly. There is already some experimental evidence of this prediction from the model [32], [33]. In 1991, Beuria & Das [32] studied the therapeutic effect of dextran sulfate on the dynamics of parasitemia in mice. Dextran sulfate is a molecule that acts by inhibiting the erythrocyte infection rate of the merozoites without killing the parasites [34]. The authors demonstrated a significant reduction of the parasitemia and better survival rates when infected mice were treated with this chemical. Although dextran sulfate cannot be used to treat human infection due to its high toxicity [32], this result supports the inhibition of the invasion of hRBC as an antimalarial target, strongly suggesting that if another substance could be identified with this effect on erythrocyte infection, but with fewer or no side effects, it could prove to be an effective antimalarial drug. Even more interestingly, Vulliez-Le Normand et al. [33] recently suggested that any therapeutic strategies aimed at inhibiting the invasion mechanism should be effective in treating malaria. These author’s findings support the idea that the interruption of the Apical Membrane Antigen 1 association with its receptor, the Rhoptry Neck Protein 2, is a target for the design and development of inhibitors. Their observations are further supported by the observation that the invasion-inhibitory peptide R1 [35], [36] blocks the interaction between the Apical Membrane Antigen 1 and the Rhoptry Neck Protein complex in *P. falciparum* [37].

The effect of increasing the death rate of gRBC, V_6_ (dividing by three the basal, reference value of γ_6_) is shown also in Figure 8 (Panel B). As a result of this intervention, it is observed that the gametocytemia is reduced and that the oscillations disappear, albeit at the expense of a small increase in merozoite load.

Figure 8C shows the result of an intervention on the third single effective target: the decrease of the activation of IS by gRBC (twofold decrease in g_73_). The aim of this intervention is to impair the stimulus exerted by the gametocytes on the immune system activation. This intervention causes a small decrease in both parasite forms (mRBC, gRBC) and an almost negligible effect on the host response (hRBC and IS).

Figure 8D shows the dynamics observed after an increase of the rate of transformation of the merozoites into gametocytes, V_5_. This was simulated through an 80-fold increase in γ_5_. What is observed are decays in mRBC and gRBC, and also a significant recovery of hRBC and IS.

Finally, Figure 8E presents the effects of the only double, simultaneous target intervention found that yielded a general improvement of the infection status. This combination involves the simultaneous decrease of the activation (dividing by two the basal, reference value of g_73_) of the rate synthesis of IS by gRBC (V_7_; the exponential effect of gRBC on IS) and the decrease (dividing by 200 the basal, reference value of g_24_) of the effect of IS over hRBC (V_2_) of the influence of IS on the recycling of hRBC. It is interesting to note that the final output is quantitatively rather similar to that observed with the single target “decreasing activation of IS by gRBC” (Panel C) but showing different dynamics.

## Discussion

The objective of this study was to identify new targets with potential for drug discovery against malaria. Since malaria is a complex disease with plenty of host–parasite interactions, the disease’s dynamics and symptoms (the system emergent properties) can only be understood through an integrated view of parasite infection and host responses. By acting on these targets, including those that only work *in vivo*, we aim to reduce the parasitemia of the two species of the parasite inside the host.

The proposed model focuses on the modeling of the underlying processes within the host-parasite dynamics of *Plasmodium* invasion of red blood cells. Its main objective is to allow for the systematic search, by means of an optimization approach**,** for the most interesting targets for drug research. Given the high number of processes involved and the model’s inherent complexity, we opted for a phenomenological representation that is simple enough to permit the proposed search but at the same time rich enough to provide valuable insight into suitable targets. Based on the results of this search, we would set up the necessary conditions to find, or help to define, therapeutic strategies, including some which are counter-intuitive. These strategies will not necessarily impede infection but will reduce parasitemia and the risk of severe symptoms as well as diminish the risk of drug resistance and selective pressure for resistant *Plasmodium* strains. The model considered a set of relevant components, processes and interactions. Variables selected for the model were the two phases of the parasite inside the host erythrocytes (mRBC, merozoites, and gRBC, gametocytes), the healthy erythrocytes of the host, hRBC, and the immune activity of the host against the parasite, IS (see Figure 1). All the variables of the model were measured in terms of their concentration inside the bloodstream. The values of the variable IS are given by the concentration of IgG1, which serves as the representative component of the mean immune response against the parasite.

We are aware of the fact that we are extrapolating the *P. chabaudi* model dynamics built from mouse data to the same process with *P. falciparum* in monkeys and humans and that *Plasmodium* life cycle lengths differ between hosts and within the erythrocyte between *Plasmodium* species. But since our approach is phenomenological, we were able to obtain a qualitative verification of the observed general pattern of behavior in monkeys and humans. The approach serves to provide information about the basic mechanism that operates in this type of infection as well as some guidance for new targets for therapeutic drugs.

The proposed model is able to describe the dynamics of the infection by *Plasmodium chabaudi* in mice. In addition, the model’s predictions were successfully compared with different experimental scenarios (Figures 3, 4 and 5). Figure 3 shows how the model reproduces the dynamics of the infection in humans after the first peak of parasitemia, predicting the attenuated oscillations and the delay observed between the phases; it also reproduces the final maintenance of the parasite load. The model’s results also correlate well with the observed response to the infection of a vaccinated host (Figure 4). The model shows the observed retardation and maximum value of the first peak of parasitemia when the host is vaccinated. Since the model is able to reproduce the disease in a third host, it can be concluded that it has captured the essentials of the host–parasite interactions. A further confirmation of the model’s reliability was obtained by comparing its predictions with the experimental measures of merozoite parasitemia in β-artemether treatment of mice infected with *Plasmodium chabaudi* [28] (Figure 5) using two different methods of inoculation of the drug. When β-artemether was injected directly into the bloodstream, the observed delayed and lower peak of parasitemia was predicted by the model (Figure 6A). Also, when the drug was inoculated through liposomes, the observed early, reduced peak of parasitemia followed by a second, lower peak was also forecast by the model (Figure 6B). Thus, the ability of the model to reproduce very different malaria infection scenarios (involving different species of *Plasmodium* as well as distinct hosts and various treatment methods) makes us confident enough to use it as a reliable tool in the search for effective pharmacological targets.

A set of such targets (single or combined) are presented in the Results section (see Figure 7). A first target would consist on decreasing the invasion rate of the erythrocytes by the merozoites; such an intervention would lead to the full recovery of the host (Figure 8A). Decreasing this rate means that free merozoites are less effective in invading red blood cells; thus they will be more exposed to the immune system [38]. Although this strategy has already been partially evaluated as a vaccination strategy [2, see also 33], our results suggest that it could also be effective as a pharmacological treatment. In fact, such an approach has been tested through the treatment of infected mice with dextran sulfate [32], a molecule that impairs the invasion of red blood cells by merozoites [34]. These authors [32] showed the effects of this substance on reducing the parasitemia and the parallel increase in the survival rate of the mice. More recently, Vulliez et al. [33] showed, through the use of structural methods, that impairment of the malaria parasite moving junction complex (decreasing the invasion rate of merozoites) is effective as antimalarial treatment. To sum up, our results indicate that decreasing the invasion rate of the red blood cells by merozoites is a target where some drug treatments can be effective against malaria.

The second single target proposed by the model works by increasing the death rate of the gametocytes. This intervention produces a decrease in gametocyte load but an increase in merozoite load, although the maximum peak will be attenuated (Figure 8B). A treatment along these lines will reduce the transmission rate during the initial stages of the disease and can be combined with traditional treatments targeting the asexual phase. Such a strategy has been already used (artemisinin combined treatments, [39]), with the predicted effects of a lower transmission rate for the disease.

A third target was to decrease the ability of the gametocytes to activate the immune system. Its effect is to reduce the maximum peaks of both parasite species (Figure 8C), and so it is expected that it would alleviate the symptoms of non-complicated malaria and reduce the transmission rate during the initial stages of the infection. This target also has additional convenient characteristics. Since it does not directly affect the parasites, there is no selective pressure on them and thus the emergence of drug resistance is avoided. Also, if combined with traditional treatments, the doses could be reduced, thereby also reducing the emergence of drug resistance (see [40]). Since this target refers to a parasite-host interaction, the only chance to observe its effects would be in *in vivo* conditions. Of equal importance is the fact that it is a very counterintuitive solution. All this could explain why this strategy has never been explored. In addition, we are aware of the technical difficulties involved in the design and implementation of such a strategy, where the sensitivity of the immune system towards the presence of one of the parasite forms is to be impaired.

The last single target identified operates by increasing the transformation rate of merozoites into envisaged gametocytes. The effect of this increase is to produce a significant decrease in merozoite load and also a decrease in gametocyte load (see Figure 8D ). Although it is not surprising that increasing the merozoite transformation rate would produce a decrease in merozoite load, what is not so intuitive is the model’s prediction of a decrease in the gametocyte load. This is a good illustration of how the integration into a mathematical representation of the many non-linear interactions among the variables involved in the relevant processes can lead to the emergence of certain counterintuitive conclusions that cannot be derived from the consideration of the local changes only. This type of intervention has been shown to relax the symptomatology of non-complicated malaria [41] and reduce transmission during the initial stages of the infection [42]. It is interesting to observe that the activation of the transformation of merozoites into gametocytes has the effect of impairing the pathogen load. A higher gametocyte growth rate yields an initial increase in its population, but this is followed by a greater decrease in such a way that, as a whole, more parasites are eliminated earlier. This behavior has been observed in other parasitic diseases [13] and can be explained by stating that a pathogen that proliferates rapidly is more likely to be detected by the immune system. Increasing the gametocytogenesis is a strategy that has never been evaluated by structural drug design methods, and our results propose this target as a promising process where these techniques can be applied for drug design.

Our exploration of the effective combinations of two targets showed that reducing the influence of gametocytes on the activation of the immune system while at the same time decreasing the effect of the immune system on the death rate of the red blood cells (see Figure 7B) would cause a reduction in both of the parasite forms (Figure 8E). The latter action can be interpreted as making the immune system less efficient in removing old erythrocytes, thus enhancing their half-life. This therapeutic strategy is optimal in order to prevent the emergence of drug resistance in the parasite. These two proposed targets have never been evaluated by structural methods. There should be great interest in examining these two processes in order to design new drugs, because affecting these processes does not generate selective pressure on the parasites; thus, effective drugs will have a low probability of leading to the emergence of resistance, thereby extending their life span. Here we can see again how the systemic approach might shed new light on a well-studied process and help propose a novel combination of targets, none of which aims to directly kill the parasites, but which could reduce the parasitemia. In all these explorations, the magnitudes of target-related parameter changes, although related with the corresponding processes, do not have a direct translation into a particular process. Accordingly, they have to be interpreted as an indication of the magnitude and sense of the intervention that would lead to the desired effect.

The new, potential antimalarial targets proposed here were identified through the use of a mathematical model that reliably reproduces the infection dynamics under different *in vivo* scenarios of the disease. This model-based strategy can be of assistance in key phases of the drug discovery process, such as the identification of the right targets, since these targets allow us to guide drug design at the molecular level through the systematic use of other means such as structural methods (molecular docking) or data-mining of inhibitor databases. A best-case illustration is provided by the proposed target consisting of decreasing the invasion of red blood cells by merozoites, which relates to the interruption of the interaction between the Apical Membrane Antigen 1 associations with its receptor, the Rhoptry Neck Protein 2. In fact, the structural details of the specific Apical Membrane Antigen 1 with the Rhoptry Neck Protein 2 interaction involved in the invasion mechanism enables the design of molecules with optimal inhibitory properties to treat malarial infection. However, due to the phenomenological character of the model, the proposed targets do not have a direct translation into a concrete, well-defined process. On the contrary, it should be stressed that the contribution of this work is to propose certain processes of the intraerythrocytic cycle of *Plasmodium* as targets for a more detailed analysis that would eventually lead to the determination of a set of concrete processes where the activity of some drugs will cause the desired global effect. This second iteration would be the natural next step in this project.

Our results suggest that the invasion of erythrocytes by merozoites is a key point where any impairment intervention would cause a decrease in parasitemia. In this case, some work has already been done with respect to structural methods of drug design [33]. Furthermore, comparison of the effect on a known drug target (β-artemether administration through liposomes; Figure 5) with the new drug target of increasing gametocytogenesis (Figure 8D) shows that both reduce the peak of parasitemia (Figure 6, panel B). When β-artemether is inoculated directly, the same peak of parasitemia is observed, but with several days’ delay (Figure 6, panel A). Chimanuka et al. [28] showed that the liposome inoculation allowed the mice to survive, in contrast with the direct inoculation. We can thus conclude that a drug which increases gametocytogenesis would produce the same effects on parasitemia (which permit survival in mice) as the β-artemether administration through liposomes.

We conclude that focusing on cellular function as a system rather than on the level of the single process or molecule will facilitate the discovery of novel classes of drugs. Through the integration into a model of the *in vivo* host–pathogen interactions, we can achieve an integrated view of parasite infection and host responses that will allow for an understanding of the host–parasite–drug interactions and the selection of drug combination strategies of therapeutic value, and for controlling the transmission of malaria by anopheline mosquitoes. In addition, the insight obtained from this work already suggests future extensions and refinements. In particular, models with a greater resolution in terms of the mechanisms involved in the novel targets such as those represented by the V_6_ (γ_6_, elimination of gRBC), V_5_, (γ_5_, gametocytogenesis), V_7_ (g_73_, activation of the immune system by the gametocytes) or V_2_ (g_24_, recycling of the erythrocytes) would lead to more mechanistically detailed versions of the model that would suggest more precise targets for drug action. We are aware of the difficulties of translating the proposed targets into precise therapies; the aim of this work is to propose targets that would direct the drug search toward certain processes of the intraerythrocytic cycle of *Plasmodium*.

Moreover, since the systemic approach used here is a general methodology, it can be used in the selection of strategies for controlling a plethora of parasitic diseases. In each case, the specific processes and their corresponding variables and parameters should be considered, but the application of the model to other forms of parasitemia would benefit from the fact that there are many common features shared by all.

## Materials and Methods

### Experimental Data

The parameters of the model were fitted using published experimental data from non-treated animal model individuals. The modelization task refers to the initial stages of the infection dynamics.

Data for the variables hRBC, mRBC and gRBC were taken from [24]. In this work, CBA/Ca mice were inoculated with 10^6^ parasitized red blood cells (*Plasmodium chabaudi*) and during the first 32 days after infection, measures were taken of the erythrocyte titer (units per µL of blood) and the parasitemia (mRBC and gRBC per µL). Data for the IS variable were taken from [25]. In this case, CBA/Ca mice were inoculated with 5·10^4^ parasitized red blood cells (*Plasmodium chabaudi*); the parasitemia dynamics of this study were compared to the parasitemia measured in [24] in order to ensure the same infection dynamics (data not shown). IgG1 from infected mice was measured during the first 17 days post infection (absorbance units). Since the dynamics of the initial stages of the variables IgG1 and IgG2A (immunoglobulin G1 and G2A) in these infected mice were almost the same (see Mota et al. 98 [25]), the IgG1 data were used to infer the mean response of the immune system against *Plasmodium*.

### Model Design

The dynamics of the infection process by *Plasmodium* were represented as a mathematical model in ordinary differential equations in the power-law formalism. In this formalism [14], which allows for non-integer kinetic orders, the processes that conforms the networks are modelled using power-law expansions in the variables of the system and are then included in non-linear ordinary differential equations with the following structure, called Generalized Mass Action (GMA):
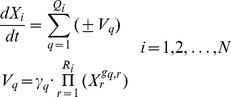
(1)


In the above expression, *X_i_* (for *i* equals 1 to N) represents the variables of the model. *V_q_* represents each of the processes affecting the variation of *X_i_*, while *X_r_* represents the variables which influence the corresponding process *V_q_*. *γ_q_* and *g_q,r_* are the parameters of the model. *γ_q_* is the rate constant related to the process *V_q_*. *g_q,r_* is the kinetic order which quantifies the effect of the variable *X_r_* on the process *V_q_*. *Q_i_* and *R_i_* are the total number of fluxes and variables, respectively.

### Model Equations

The model derived from the scheme in Figure 1 is given by:
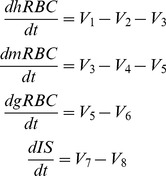
(2)where the corresponding rate equations in power law form corresponding to the different fluxes are:



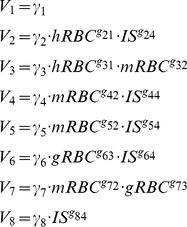
(3)
Table 1 shows the values of the model parameters: This set of value corresponds to the only solution which fitted the data and verifies new experimental conditions (see results). Figure 2 presents the corresponding model data fitting.

**Table 1:** Model parameter values.

Rate constants		Kinetic orders			
Name	Value	Name	Value	Name	Value
**γ_1_**	0.97	**g_21_**	3.00	**g_63_**	0.84
**γ_2_**	0.94	**g_24_**	1.73	**g_64_**	0.83
**γ_3_**	0.51	**g_31_**	1.52	**g_72_**	0.22
**γ_4_**	0.73	**g_32_**	1.01	**g_73_**	0.63
**γ_5_**	0.26	**g_42_**	0.84	**g_84_**	4.29
**γ_6_**	0.49	**g_44_**	2.98		
**γ_7_**	0.15	**g_52_**	1.80		
**γ_8_**	0.09	**g_54_**	2.98		

### Parameter Estimation and Model Fitting

Model parameters were determined by fitting the model to experimental data from infected mice during the first month of the disease after infection [24], [25] (see Figure 2). The fitting was attained through the use of a heuristic optimization algorithm (Modified Genetic Algorithm) previously used and presented by us elsewhere [43]. The objective function (*Fobj*) is minimized in the process:
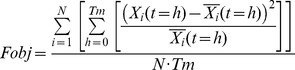
(4)


In this equation, 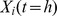 is the *X_i_* variable value at the time point *h* and 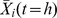 is the corresponding experimental value at the same time point *h*. The stopping criterion is the maximum number of iterations of the algorithm (1000), which in this model satisfy the convergence of the majority of the solutions. The best minimum reached is expected to reproduce the qualitative dynamic behavior of the measured data. Table 2 shows the value of the objective function of all the solutions presented. Values much higher than one (in days units) for γ parameters in a cellular scale would correspond to an excessive fast processes, furthermore values of g’s higher than 3 represent strong sensitivities of the processes by the variables. Because of that, the parameter search boundaries were [0, 1] for the γ’s, [0, 3] for the g’s and [0, 6] for g_84_. In this last case, this was due to the fact that g_84_ represents the combined influence on V_8_ of IS as substrate and effector.

**Table 2:** Objective functions.

Fopt_both_				Fopt_g_	
Sol_ori_	0.408	Sol_γ5_	0.202	Sol_ori_	0.490
Sol_γ3_	0.023	Sol_g73_	0.289	Sol_g73/g24_	0.202
Sol_γ6_	0.271				

*So_ori_* is the value of the objective function of the solution represented in Figure 2. *Sol_γ3_*, *Sol_γ5,_ Sol_γ6,_ Sol_g73,_ and Solu_g73/g24_* are the values of the objective functions for the solutions corresponding to the pharmacological treatment in γ_3_, γ_5_, γ_6_, g_73_ and the combination of g_73_ and g_24_ respectively (see Figure 7). *Fobj_both_* and *Fobj_g_* are described in Material and Methods (equations 6).

### Model Validation

Any useful model is expected to reproduce the observed responses against different experimental scenarios and treatments. To do so here, we used other experimental data obtained in conditions of pharmacological treatment or from vaccinated individuals.

The first model validation comes from the comparison of the model dynamics of the parasite variables with the corresponding experimental values in malaria-affected humans [26]. A second set of comparisons was obtained from data taken from vaccinated and non-vaccinated infected monkeys [27]. In this case, the parasitemia course observed after the vaccination was used to verify the model response under a simulated vaccination. This last condition was performed in the model by increasing the initial value of the IS variable. The last verification test was realized by comparing the model’s results against experimental data obtained from treated and non-treated mice infected with *Plasmodium* [28]. Two different forms of drug administration were considered: direct drug injection (*Free drug*) and administration through liposomes (*Liposome drug*). Equation 5 presents the model equations corresponding to these two modes of drug administration (see Figures 6A and 6B).

Direct drug injectionLiposome drug administration.
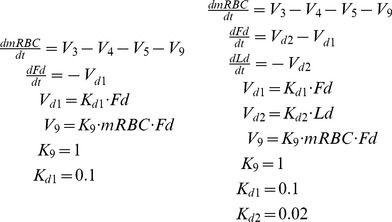
(5)


In these equations, *Fd* represents the variable Free drug (drug in the bloodstream), *Ld* represents the variable Liposome drug (drug inside liposomes), and *V_d1_* is the drug clearance from the body in both the free drug and liposome drug administration models. *K_d1_* and *K_d2_* are the corresponding rate constants and *V_d2_* is the drug diffusion from the liposomes. The parameter values K_9_, K_d1_ and K_d2_ were estimated in order to fit the time scale and the relative decrease of the peak of parasitemia.

### Search for Pharmacological Targets

In order to select the targets, each model parameter linked to a potential target was changed either one at a time or in combination with another, in an attempt to identify solutions showing reduced objective function. The objective function considers the relative difference of the mean healthy red blood cells with respect to the initial non-infected condition and the relative increase in the maximum parasite peak and the mean parasite load with respect to the initial value of parasites. So the search consists of looking for solutions with low values of objective function, which will be closer to the healthy situation. This set of parameter values was selected as the proposed target. For the combined targets, we chose those which were better than the worst of the single targets. The objective functions used during the search are displayed in Equation 6.
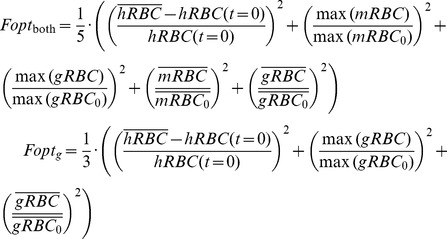
(6)


*Fopt_both_* is the objective function used to select solutions having low levels in both forms of the parasite and healthy levels of erythrocytemia. The first term of this equation represents the relative squared difference between the mean value of the hRBC variable (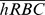) of the solution and the value of this variable under healthy conditions (*hRBC(t = 0)*), which has to be minimized for obtaining solutions with healthy levels of non-infected red blood cells. The second and third terms represent the relative value of the maximum level of parasitemia with respect to the initial value of the parasitemia; minimizing these allows us to obtain solutions with attenuated peaks of parasitemia. Finally, the fourth and fifth terms represent the relative value of the mean level of parasitemia during the infection with respect to the initial value of parasitemia; minimizing these allows us to obtain solutions with low levels of parasitemia. *Fopt_g_* was used to find solutions with low gametocytemia. As can be seen in this equation, the three terms used correspond to those terms of the previous equations that are related to the gRBC species, because this objective function only considers the minimization of the gametocyte. Overlined variables represent the mean value of the corresponding variables over time; the subscript 0 represent the initial value, before the optimization. In both cases, the sum is divided by the number of summands (5 and 3 respectively) in order to compare the values of the objective function. Table 2 shows the value of the objective function for all the proposed targets.

## Supporting Information

Figure S1 **Absolute values of the steady state sensitivities at the healthy condition.**
(TIF)

Figure S2 **Absolute values of the dynamic sensitivities.**
(TIF)

Information S1 **Steady State Stability and Sensitivity Analysis.**
(DOCX)
