# Supplementary figures and images for: New Targets for Drug Discovery against Malaria
Source: PLoS One. 2013 Mar 28;8(3):e59968. doi: 10.1371/journal.pone.0059968 (PMC3610898; doi:10.1371/journal.pone.0059968)

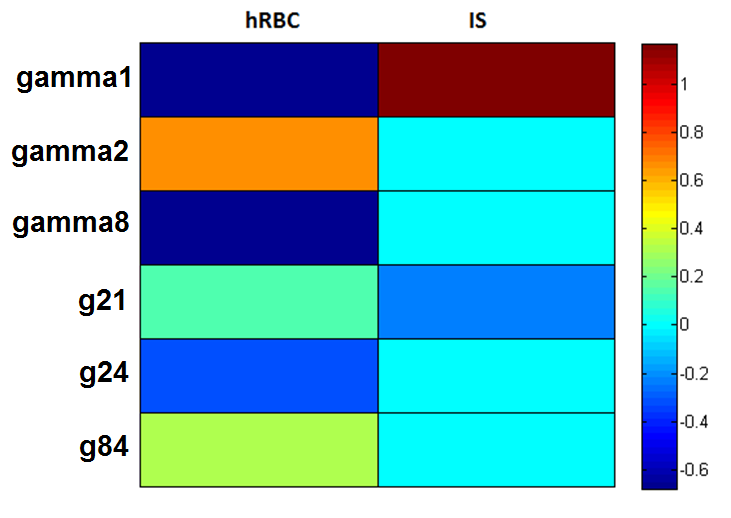

Supplement: Figure S1 — Absolute values of the steady state sensitivities at the healthy condition. (TIF) [file pone.0059968.s001.tif]

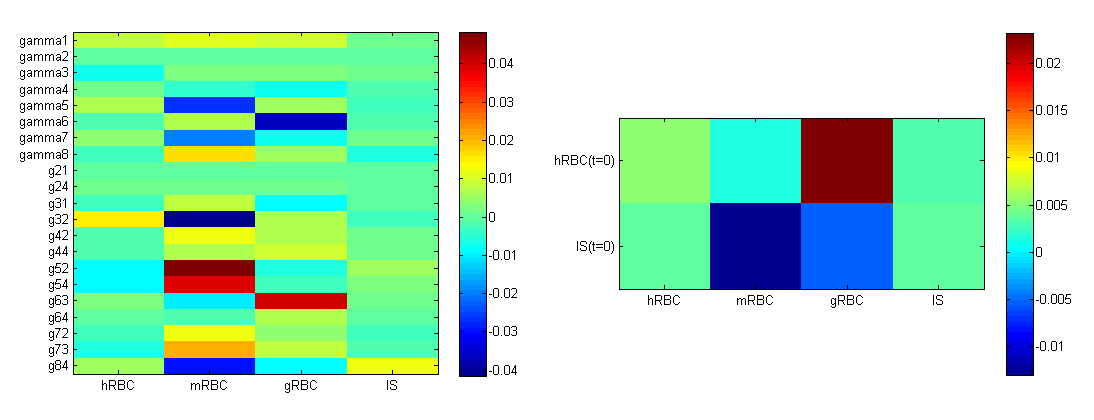

Supplement: Figure S2 — Absolute values of the dynamic sensitivities. (TIF) [file pone.0059968.s002.tif]
