# Supplementary material for: New Targets for Drug Discovery against Malaria
Source: PLoS One. 2013 Mar 28;8(3):e59968. doi: 10.1371/journal.pone.0059968 (PMC3610898; doi:10.1371/journal.pone.0059968)
Supplement: Information S1 — Steady State Stability and Sensitivity Analysis. (DOCX) [file pone.0059968.s003.docx]

**Information S1. Steady State Stability and Sensitivity Analysis.**

Any mathematical model of a biological system has to be stable and robust if it is to be considered a reliable representation of the real system. The model was thus submitted to a stability and sensitivity analysis. We determined that the healthy host steady state is stable (see Supporting Information, Section 3). At the same time, in order to check the model’s robustness, we evaluated two types of system sensitivities: the steady state sensitivities, which refer to the normal, healthy steady state, and the dynamic system sensitivities parameters, which identify the parameters with major influence on the transient dynamics (see Supporting Information, Section 4). From this analysis we concluded that the model is robust enough to represent the biological system.

1. **Stability Analysis**

The stability of the healthy, reference steady state can be found in the model shown in Equation S1 that corresponds to the not infected, healthy host steady state through the evaluation of eigenvalues of the corresponding jacobian matrix:

$\begin{matrix} \frac{dhRBC}{dt}=V_{1}-V_{2} \\ \frac{dIS}{dt}=V_{IS}-V_{8} \\ V_{1}=\gamma_{1} \\ V_{2}=\gamma_{2}\cdot hRBC^{g_{21}}\cdot IS^{g_{24}} \\ V_{8}=\gamma_{8}\cdot IS^{g_{84}} \\ V_{IS}\approx0 \end{matrix}$ (Equation S1)

where hRBC and IS are the same as in the original model (Equations 2), and *V_IS_* is the rate associated with the host variable IS in the absence of infection.

1. **Sensitivity Analysis**

Sensitivity analysis enables the identification of parameters that exert a major influence on system response. Since we depart from and aim to reach a steady state where the variable values are stable at normal, healthy values, we should evaluate the robustness of this reference steady state. But our model is a dynamic one, too; therefore, it is also necessary to identify the parameters with a major influence on the transient dynamics.

*Steady state sensitivities.* Steady state sensitivities were calculated at the healthy, not infected steady state [1, 2, 3]. Steady state sensitivities measure the relative change to the value of variables with respect to an infinitesimal change in the parameters (kinetic orders and rate constants) or in the initial conditions. These sensitivities were calculated in accordance with Equation S2.

******

******

(Equation S2)

In the above expressions, *SS(X_i_,P_k_)* and *SS(X_i_,X_j_(t=0))* are the sensitivities of the variable *X_i_* with respect to changes in parameter *P_k_* and initial condition *X_j_(t=0)*, respectively; *sX_i_* is the steady state value of the variable *X_i_*; *X_j_(t=0)* is the initial condition value of the variable *X_j_* and *P_k_* is the value of the parameter *k*. Carets are displayed over the variables and parameters which correspond to the perturbed situation. Figure 9 shows the values of the steady state sensitivity at the healthy condition when the variables mRBC and gRBC are not present. In all cases, the maximum absolute value of sensitivities is about 1.2.

Figure S1

**
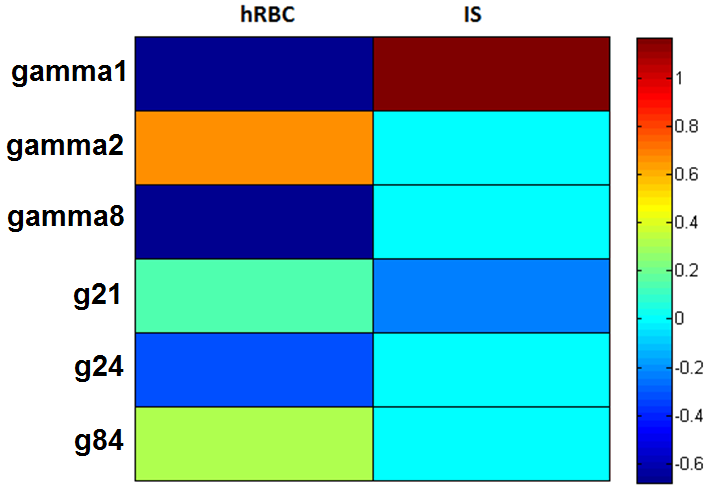
**

*Dynamic sensitivities.* Dynamic sensitivities measure the relative change on the value of the area under the curve of all the variables with respect to an infinitesimal change in the parameters (kinetic orders and rate constants) or in the initial conditions [see 4]. For this purpose we used the following equations:

$i=1,2,\ldots,N;k=1,2,\ldots,M$

(Equation S3)

In the above expressions, *DS(X_i_,P_k_)* and *DS(X_i_,X_j_(t=0))* are the dynamic sensitivities of the variable *X_i_* with respect to changes in parameter *P_k_* and initial condition *X_j_(t=0)*, respectively; $\int_{t=0}^{\mathrm{Tm}} X_{i}\mathrm{dt}$represents the area under the curve of the variable *X_i_* during the time between 0 and final time *Tm*. Carets are displayed over the variables and parameters which correspond to the perturbed situation. Figure
S2 shows the values of the dynamic sensitivities. In all cases the maximum absolute value of sensitivities is about 1.2.

Figure S2

**
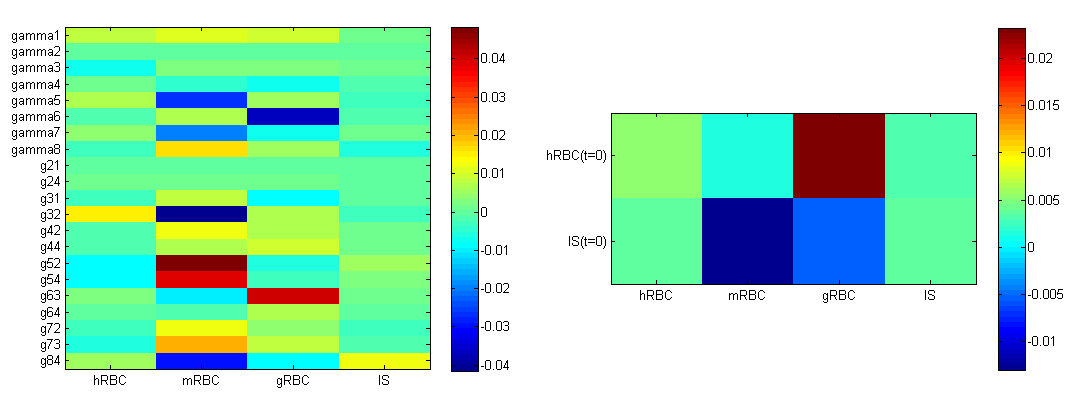
**

**References**

1 Voit EO (2000) Computational analysis of biochemical systems*.* Cambridge University Press.

2 Siljak DD (1969) Nonlinear system: The parameter analysis and design. New York: Wiley.

3 Frank PM (1978) Introduction to systems sensitivity theory. New York: Academic Press.

4 Hormiga J (2010) Quantitative analysis of the dynamic signaling pathway involved in the cAMP mediated induction of L-carnitine biosynthesis in E. coli cultures. Molecular Biosystems *6*(4): 699-710.
